# Modifiable and unmodifiable factors associated with slow flow following rotational atherectomy

**DOI:** 10.1371/journal.pone.0250757

**Published:** 2021-04-26

**Authors:** Kenichi Sakakura, Yousuke Taniguchi, Kei Yamamoto, Takunori Tsukui, Hiroyuki Jinnouchi, Masaru Seguchi, Hiroshi Wada, Hideo Fujita

**Affiliations:** Division of Cardiovascular Medicine, Saitama Medical Center, Jichi Medical University, Saitama City, Japan; Baylor Scott and White, Texas A&M College of Medicine, UNITED STATES

## Abstract

**Background:**

Although several groups reported the risk factors for slow flow during rotational atherectomy (RA), they did not clearly distinguish modifiable factors, such as burr-to-artery ratio from unmodifiable ones, such as lesion length. The aim of this retrospective study was to investigate the modifiable and unmodifiable factors that were associated with slow flow.

**Methods:**

We included 513 lesions treated with RA, which were classified into a slow flow group (n = 97) and a non-slow flow group (n = 416) according to the presence or absence of slow flow just after RA. The multivariate logistic regression analysis was performed to find factors associated with slow flow.

**Results:**

Slow flow was inversely associated with reference diameter [Odds ratio (OR) 0.351, 95% confidence interval (CI) 0.205–0.600, *p*<0.001], primary RA strategy (OR 0.224, 95% CI 0.097–0.513, *p*<0.001), short single run (≤15 seconds) (OR 0.458, 95% CI 0.271–0.776, *p* = 0.004), and systolic blood pressure (BP) ≥ 140 mmHg (OR 0.501, 95% CI 0.297–0.843, *p* = 0.009). Lesion length (every 5 mm increase: OR 1.193, 95% CI 1.093–1.301, *p*<0.001), angulation (OR 2.054, 95% CI 1.171–3.601, *p* = 0.012), halfway RA (OR 2.027, 95% CI 1.130–3.635, *p* = 0.018), initial burr-to-artery ratio (OR 1.451, 95% CI 1.212–1.737, *p*<0.001), and use of beta blockers (OR 1.894, 95% CI 1.004–3.573, *p* = 0.049) were significantly associated with slow flow.

**Conclusions:**

Slow flow was positively associated with several unmodifiable factors including lesion length and angulation, and inversely associated with reference diameter. In addition, slow flow was positively associated with several modifiable factors including initial burr-to-artery ratio and use of beta blockers, and inversely associated with primary RA strategy, short single run, and systolic blood pressure just before RA. Application of this information could help to improve RA procedures.

## Introduction

Severe calcification in coronary artery disease is strongly associated with poor outcomes following percutaneous coronary intervention (PCI) [1–3]. Rotational atherectomy (RA) has been a reliable procedure for the treatment of severely calcified coronary lesions for more than 20 years [4, 5]. However, unique complications such as slow flow, vessel perforation, and burr entrapment can occur during RA [6–10], which accounts for a greater incidence of severe outcomes after PCI with RA compared to that without RA [11]. Among complications in RA, slow flow is the most common [12]. Although several groups reported risk factors for slow flow [13, 14], they did not clearly distinguish modifiable factors such as burr-to-artery ratio from unmodifiable factors such as lesion length. It is important for the refinement of RA procedures to understand the modifiable factors, and important also for accurate risk assessment to understand those that are unmodifiable.

Unlike vessel perforation and burr entrapment, slow flow following RA is a transient phenomenon in most cases. Accordingly, large multi-center registries as well as retrospective single-center cohort studies might not accurately record the occurrence of transient slow flow. In fact, the incidence of slow flow following RA was much lower than that of periprocedural myocardial infarction (MI) following RA in several retrospective studies (1.1% vs. 7.4% [15], 0.0% vs. 14.0% [16], 2.6% vs. 6.9% [17]), which is uncommon in clinical practice. On the other hand, since prospective studies regarding the incidence of slow flow focused on specific topics, such as drug cocktails or rotational speed [14, 18], analyses to find determinants of slow flow were not adequately performed. Therefore, there remains an unmet need to identify modifiable and unmodifiable factors associated with slow flow using a large database that recorded slow flow accurately. The aim of this retrospective study was to investigate modifiable and unmodifiable factors associated with slow flow accurately recorded.

## Methods

### Study patients

This was a retrospective, single-center study. We reviewed PCI reports during the period from November 2014 to August 2020. The inclusion criteria were (1) PCIs that were performed in the catheter laboratory in the Saitama Medical Center, Jichi Medical University, and (2) PCIs in which RA was used. The exclusion criteria were (1) PCIs without RA and (2) coronary flow was not confirmed immediately just after RA. Indications for RA in our institution are the following: 1) angiographically moderate or severely calcified lesions, 2) diffuse lesions expected to be difficult to stent, and 3) ostial lesions [12, 14].

**Fig 1:**
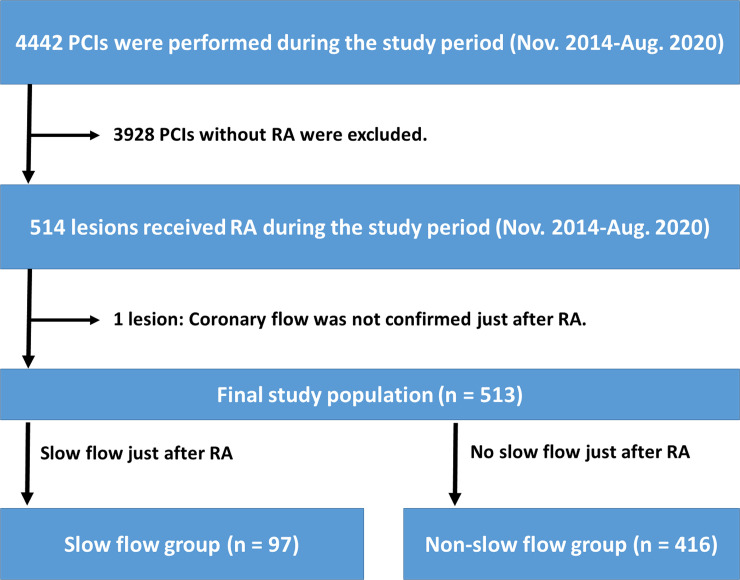
Study flow chart. Abbreviations: PCI = percutaneous coronary intervention, RA = rotational atherectomy.

During the study period, a total of 4442 PCIs were performed. Of 4442 PCIs, 3928 PCIs without RA were excluded. Of 514 PCIs with RA, one lesion was excluded, because coronary flow was not confirmed immediately after RA. Except for this lesion, we routinely checked coronary flow just after RA in all lesions, partly because our group conducted a prospective randomized study regarding the slow flow [14]. In the randomized study, we compared the incidence of slow flow between low-speed group (140,000 rpm) and high-speed group (190,000 rpm) using 100 patients from November 2014 to February 2016 [14]. The present study did not exclude those patients. The final study consisted of 513 lesions. The lesions were classified into a slow flow group (n = 97) and a non-slow flow group (n = 416) according to the presence or absence of slow flow being defined as transient thrombolysis in myocardial infarction (TIMI) flow grade ≤2 just after RA [19]. The evaluation of TIMI flow grade just after RA was performed by an unblinded operator (K. Sakakura). The study flow chart is shown in Fig 1.

The study was approved by the institutional review board of the Saitama Medical Center, Jichi Medical University (S20-084), and written informed consent was waved by the institutional review board of the Saitama Medical Center, Jichi Medical University, because of the retrospective study design. All methods were performed in accordance with the ethical guidelines of the 1975 Declaration of Helsinki.

In the Saitama Medical Center, Jichi Medical University, the annual average number of all PCI procedures were approximately 700–800 per year. We had 2 catheter rooms for PCI, and several staff interventional cardiologists (K. Sakakura, Y. Taniguchi, K. Yamamoto, T. Tsukui, H. Jinnouchi, and M. Seguchi), which was not consistent during the study period. Our medical center started RA more than 15 years ago. During the study period, most RA was performed or supervised by a senior interventional cardiologist (K. Sakakura).

In most cases, RA was selected as the primary RA strategy, defined as RA before any attempt of balloon dilatation, whereas in some cases RA was selected as the secondary RA strategy, defined as RA after unsuccessful balloon dilatation or unsuccessful balloon delivery. In the primary RA strategy, RA was performed using standard techniques. Intravenous heparin was used to achieve an appropriate activated coagulation time (≥250 seconds). We did not use any glycoprotein IIb/IIIa inhibitors, which were not available in Japan. We used the nicorandil based drug cocktail (nicorandil 12 mg, isosorbide dinitrate 2.5 mg, heparin 10,000 units, and normal saline 500 mL) for all RA cases. The lesion was crossed with a 0.014-inch conventional guidewire. Of 513 lesions, intravascular imaging including intravascular ultrasound (IVUS) or optical coherent tomography (OCT) before RA was tried in 356 lesions (69.4%). Of 356 lesions, intravascular imaging devices crossed the lesion before RA in 192 lesions (37.4%). After intravascular imaging, a 0.014-inch conventional guidewire was exchanged for a 0.009-inch RotaWire floppy or RotaWire extra support guidewire (Boston Scientific, Marlborough, MA, USA) using a microcatheter. The RA burr was subsequently advanced over the wire to a position proximal to the lesion. Blood pressure (BP) and heart rate were recorded immediately before RA. The initial rotational speed was set within the conventional range (140,000–190,000 rpm) with the burr proximal to the lesion. Of 513 lesions, 100 lesions from November 2014 to February 2016 were randomly allocated to 140,000 rpm or 190,000 rpm [14]. The burr was activated and moved forward with a slow pecking motion. Each run time was <30 seconds, and care was taken to avoid a decrease in rotational speed >5000 rpm. However, excessive speed down was sometimes observed especially in the ostium of the right coronary artery [20]. The initial burr size was either 1.25-mm, 1.5-mm, or rarely 1.75-mm. After the burr passed the lesion, the burr was removed using the dynaglide mode or trapping balloon technique [21]. The presence of coronary flow was confirmed by injecting sufficient contrast medium immediately after the burr had been removed. Following RA, balloon dilatation was performed using a non-compliant balloon/scoring balloon/cutting balloon to facilitate stent implantation. In selected cases, we performed halfway RA. Halfway RA is a strategy, in which an operator does not advance the burr to the end of a continuous calcified lesion, and performs balloon dilatation to treat the remaining part of the calcified lesion [22]. Halfway RA was typically performed to severely angulated lesions [22, 23]. An intra-aortic balloon pump (IABP) was inserted via a femoral artery before RA in high-risk cases such as those with severe left ventricular dysfunction, unprotected left main stenosis, or severe 3-vessel disease. This was done because complications such as slow flow or peri-procedural MI have been shown to be more frequent in these high-risk cases [12].

We collected data on the following complications: slow flow just after RA, vessel perforation (type III) due to the burr, burr entrapment, and periprocedural MI with slow flow. Peri-procedural MI was defined as an increase in creatine kinase (at least three-fold above the normal upper limit) [12, 14]. Hypertension was defined as a systolic BP > 140 mmHg, diastolic BP > 90 mmHg, or medical treatment for hypertension [14]. Diabetes mellitus was defined as a hemoglobin A1c level > 6.5% or treatment for diabetes mellitus [14, 24]. Hyperlipidemia was defined as a total cholesterol level > 220 mg/dl, a low-density lipoprotein cholesterol level > 140 mg/dl, or treatment for hyperlipidemia [14]. eGFR was calculated using the MDRD formula [25]. ACS was defined as ST-segment elevation MI, non-ST-segment elevation MI, or unstable angina [14]. Left ventricular ejection fraction (LVEF) was obtained from the official echocardiography report within 3 months before RA. Although LVEF values measured by the modified Simpson’s method was used for this study, LVEF values measured by the Teichholz method was adopted when LVEF values of modified Simpson’s method were not available. The reference diameter and lesion length were calculated by quantitative coronary angiography. Offline, computer-based software QAngio XA 7.3 (MEDIS Imaging Systems, Leiden, The Netherlands) was used for quantitative coronary angiography. Calcification was identified as readily apparent radiopacities within the vascular wall at the site of the stenosis, and was classified as severe (radiopacities noted without cardiac motion before contrast injection generally compromising both sides of the arterial lumen) [26]. The burr-to-artery ratio was defined as the burr size divided by the reference diameter.

### Statistical analysis

Data are presented as a percentage for categorical variables, a mean ± SD for normally-distributed continuous variables, or a median and inter-quartile range for non-normally-distributed continuous variables. The Wilk-Shapiro test was performed to determine if the continuous variables were normally distributed. Normally distributed continuous variables were compared between the 2 groups using Student’s t-test. Otherwise, continuous variables were compared using a Mann-Whitney U test. Categorical data were compared using Fischer’s exact test. We performed multivariate logistic regression analysis to investigate factors associated with slow flow. First, we performed two analyses for modifiable and unmodifiable factors separately. In both analyses, the dependent variable was slow flow just after RA. Variables that had a marginal difference (*p* <0.20) between the 2 groups were used as independent variables. However, similar variables such as initial burr-to-artery ratio and final burr-to-artery ratio were not entered into the model simultaneously to avoid multicollinearity. Moreover, the variables with many missing values were not entered into the model. Then, we made a final model to investigate the association between slow flow and modifiable/unmodifiable factors. In this model, variables that had a significant association (*p* <0.05) in each multivariate logistic regression analysis were included as independent variables. We did not enter all marginally associated variables into a single model, because the number of events per variable should be less than 10 [27, 28]. Odds ratios (OR) and the 95% confidence intervals (CI) were calculated. All reported P-values were determined by two-sided analysis, and *p*-values <0.05 were considered significant. The data obtained were entered into Microsoft Excel (Redmond, WA, USA), and all analyses were performed with IBM SPSS statistics version 25 (Chicago, IL, USA).

## Results

The comparisons of the patients and lesion characteristics between the 2 groups are summarized in Table 1. The reference diameter was significantly smaller in the slow flow group than in the non-slow flow group. The lesion length was significantly greater in the slow flow group than in the non-slow flow group. Moderate to severe angulation was more frequently observed in the slow flow group. The comparison of procedural characteristics between the 2 groups is summarized in Table 2. Primary RA strategy was less frequently adopted in the slow flow group than in the non-slow flow group. Initial burr-to-artery ratio was significantly greater in the slow flow group than in the non-slow flow group. Mean single run time was significantly longer in the slow flow group. Systolic BP just before RA was significantly lower in the slow flow group. The overall incidence of peri-procedural MI with slow flow, final TIMI flow grade ≤2, vessel perforation due to burr, and burr entrapment was 1.9%, 0.8%, 0.4%, and 0.2%, respectively.

**Table 1:** Comparison of patients and lesions characteristics between the slow flow group and non-slow flow group.

	All	Slow flow group	Non-slow flow group	*p* value
	(n = 513)	(n = 97)	(n = 416)	
Patient characteristics				
Age (years)	75 (69–79)	76 (69–80)	75 (69–79)	0.808
Men—n, (%)	381 (74.3)	73 (75.3)	308 (74.0)	0.898
Overweight (BMI ≥25 kg/m^2^)—n, (%)	136 (26.5)	24 (24.7)	112 (26.9)	0.703
Hypertension—n, (%)	494 (96.3)	95 (97.9)	399 (95.9)	0.550
Diabetes mellitus—n, (%)	286 (55.8)	61 (62.9)	225 (54.1)	0.140
Hyperlipidemia—n, (%)	478 (93.2)	90 (92.8)	388 (93.3)	0.825
Current smoker—n, (%) (n = 511)	84 (16.4)	16 (16.7)	68 (16.4)	1.000
Chronic renal failure (creatinine >2mg/dl)—n, (%)	136 (26.5)	21 (21.6)	115 (27.6)	0.252
Estimated GFR (mL/min/1.73m^2^)	76.5 (22.0–100.8)	76.3 (49.0–100.1)	76.5 (17.1–101.3)	0.738
Left ventricular ejection fraction (%)	61.0 (50.0–68.0)	60.3 (45.6–65.7)	61.0 (50.7–68.0)	0.125
	(n = 377)	(n = 67)	(n = 310)	
Chronic renal failure on hemodialysis—n, (%)	121 (23.6)	15 (15.5)	106 (25.5)	0.046
History of hospitalization caused by heart failure–n, (%)	114 (22.2)	22 (22.7)	92 (22.1)	0.893
Statin treatment—n, (%)	468 (91.2)	89 (91.8)	379 (91.1)	1.000
ACE inhibitors/ARBs treatment—n, (%)	326 (63.5)	70 (72.2)	256 (61.5)	0.061
Beta blockers treatment—n, (%)	371 (72.3)	78 (80.4)	293 (70.4)	0.058
Lesion characteristics				
Culprit lesion in acute coronary syndrome—n, (%)	94 (18.3)	21 (21.6)	73 (17.5)	0.382
Culprit lesion in acute coronary syndrome with visible thrombus- n, (%)	2 (0.4)	1 (1.0)	1 (0.2)	0.343
Chronic total occlusion–n, (%)	10 (1.9)	4 (4.1)	6 (1.4)	0.100
In-stent lesion–n, (%)	28 (5.5)	2 (2.1)	26 (6.3)	0.135
Target coronary artery				0.444
Left main- left anterior descending artery—n, (%)	361 (70.4)	69 (71.1)	292 (70.2)	
Left circumflex artery—n, (%)	30 (5.8)	3 (3.1)	27 (6.5)	
Right coronary artery—n, (%)	122 (23.8)	25 (25.8)	97 (23.3)	
Specific target coronary artery				
Ostial left main–n, (%)	4 (0.8)	0 (0)	4 (1.0)	1.000
Ostial left anterior descending artery–n, (%)	59 (11.5)	15 (15.5)	44 (10.6)	0.214
Ostial left circumflex artery–n, (%)	8 (1.6)	0 (0)	8 (1.9)	0.363
Ostial right coronary artery—n, (%)	36 (7.0)	8 (8.2)	28 (6.7)	0.658
Any ostial lesion–n, (%)	107 (20.9)	23 (23.7)	84 (20.2)	0.488
Reference diameter (mm)	2.33 (1.97–2.77)	2.04 (1.74–2.36)	2.42 (2.04–2.83)	<0.001
Lesion length (mm)	21.87 (11.89–34.48)	33.41 (19.45–47.04)	19.54 (10.50–31.87)	<0.001
Lesion angle				<0.001
Mild angulation (<30°)	269 (52.4)	29 (29.9)	240 (57.7)	
Moderate angulation (30-60°)	189 (36.8)	47 (48.5)	142 (34.1)	
Severe angulation (≥60°)	55 (10.7)	21 (21.6)	34 (8.2)	
Angiographically severe calcification	505 (98.4)	95 (97.9)	410 (98.6)	0.650
Pre-procedural TIMI-flow grade ≤2	61 (11.9)	24 (24.7)	37 (8.9)	<0.001

Data are expressed as median and inter-quartile range or number (percentage). A Mann-Whitney U test was used for continuous variables, and a Fischer exact test was used for categorical variables. Abbreviations: GFR = glomerular filtration rate, TIMI = Thrombolysis in myocardial infarction, ACE inhibitors = angiotensin converting enzyme inhibitors, ARBs = angiotensin II receptor blockers.

**Table 2:** Comparison of procedural characteristics and outcomes between the slow flow group and non-slow flow group.

	All	Slow flow group	Non-slow flow group	*p* value
	(n = 513)	(n = 97)	(n = 416)	
Primary RA strategy–n, (%)	467 (91.0)	76 (78.4)	391 (94.0)	<0.001
Guiding catheter size and system				0.789
6Fr—n, (%)	9 (1.8)	1 (1.0)	8 (1.9)	
7Fr—n, (%)	468 (91.2)	88 (90.7)	380 (91.3)	
8Fr—n, (%)	36 (7.0)	8 (8.2)	28 (6.7)	
Intra-aortic balloon pump support—n, (%)	37 (7.2)	11 (11.3)	26 (6.3)	0.085
Guidewire used during rotational atherectomy				<0.001
RotaWire floppy—n, (%)	402 (78.4)	60 (61.9)	342 (82.2)	
RotaWire extra support—n, (%)	75 (14.6)	20 (20.6)	55 (13.2)	
Guidewire switch from floppy to extra support—n, (%)	31 (6.0)	16 (16.5)	15 (3.6)	
Guidewire switch from extra support to floppy–n, (%)	5 (1.0)	1 (1.0)	4 (1.0)	
Number of burrs used	1 (1–1)	1 (1–1)	1 (1–1)	0.222
Initial burr size				0.063
1.25-mm	212 (41.3)	50 (51.5)	162 (38.9)	
1.5-mm	298 (58.1)	47 (48.5)	251 (60.3)	
1.75-mm	3 (0.6)	0 (0)	3 (0.7)	
Final burr size				0.080
1.25-mm	199 (38.8)	48 (49.5)	151 (36.3)	
1.5-mm	246 (48.0)	40 (41.2)	206 (49.5)	
1.75-mm	27 (5.3)	5 (5.2)	22 (5.3)	
2.0-mm	41 (8.0)	4 (4.1)	37 (8.9)	
Initial burr-to-artery ratio	0.60 (0.51–0.70)	0.69 (0.58–0.80)	0.58 (0.50–0.69)	<0.001
Final burr-to-artery ratio	0.62 (0.53–0.72)	0.69 (0.58–0.82)	0.61 (0.52–0.70)	<0.001
Total run time (seconds)	77.0 (47.5–117.0)	116.0 (67.0–185.5)	73.0 (45–108)	<0.001
Mean single run time (seconds)	12.8 (10.8–16.8)	14.8 (11.6–18.6)	12.7 (10.7–16.1)	<0.001
Short single run (mean single run time ≤15 seconds)–n, (%)	340 (66.3)	50 (51.5)	290 (69.7)	0.001
Mean rotational speed (x 1000 rpm)	176.4 (160.0–179.5)	177.7 (168.5–180.2)	176.3 (159.7–179.2)	0.059
Maximum speed reduction during RA (rpm) (n = 506)	5000 (4000–8000)	6000 (5000–9000)	5000 (4000–7000)	<0.001
Systolic blood pressure just before RA (mm Hg)	150 (132–168)	141 (131–159)	152 (134–170)	0.002
Diastolic blood pressure just before RA (mm Hg)	75 (67–84)	73 (63–80)	76 (68–85)	0.014
Heart rate just before RA (per minute)	70 (62–78)	69 (62–78)	70 (61–78)	0.921
Halfway RA–n, (%)	103 (20.1)	36 (37.1)	67 (16.1)	<0.001
Final procedure				0.161
RA + balloon including drug-coating balloon—n, (%)	32 (6.2)	5 (5.2)	27 (6.5)	
RA + bare-metal stent—n, (%)	7 (1.4)	0 (0)	7 (1.7)	
RA + drug-eluting stent—n, (%)	471 (91.8)	90 (92.8)	381 (91.6)	
RA + covered stent for perforation—n, (%)	2 (0.4)	1 (1.0)	1 (0.2)	
Unsuccessful revascularization–n, (%)	1 (0.2)	1 (1.0)	0 (0)	
Complications and outcomes				
Periprocedural MI with slow flow–n, (%)	10 (1.9)	10 (10.3)	0 (0)	<0.001
Final TIMI flow grade ≤2 –n, (%)	4 (0.8)	4 (4.1)	0 (0)	0.001
Vessel perforation (Type III) due to burr–n, (%)	2 (0.4)	1 (1.0)	1 (0.2)	0.343
Slow flow requiring VA-ECMO–n, (%)	2 (0.4)	2 (2.1)	0	0.035
Aortic cusp dissection–n, (%)	1 (0.2)	0	1 (0.2)	1.000
Burr entrapment–n, (%)	1 (0.2)	0 (0)	1 (0.2)	1.000
In-hospital death (irrespective of procedural complications)	7 (1.4)	2 (2.1)	5 (1.2)	0.622

Data are expressed as median and inter-quartile range or number (percentage). A Mann-Whitney U test was used for continuous variables, and a Fischer exact test was used for categorical variables. Abbreviations: RA = rotational atherectomy, GFR = glomerular filtration rate, VA-ECMO = veno-arterial extracorporeal membrane oxygenation.

Multivariate logistic regression analysis to investigate the association between unmodifiable factors and slow flow is shown in Table 3. Among 8 variables, reference diameter was inversely associated with slow flow. Lesion length, moderate to severe angulation (≥30°), and pre-procedural TIMI-flow grade ≤2 were significantly associated with slow flow. The multivariate logistic regression analysis to investigate the association between modifiable factors and slow flow is shown in Table 4. Among 7 variables, primary RA strategy, and systolic BP just before RA were inversely associated with slow flow. Initial burr-to-artery ratio, mean single run time, and halfway RA were significantly associated with slow flow.

**Table 3:** Multivariate logistic regression model to find unmodifiable factors associated with slow flow.

Dependent variable: Slow flow (≤ TIMI-2) just after RA			
*Dependent variable*: *Slow flow*			
*Independent variables*	Odds ratio	95% confidence interval	*p* value
Diabetes mellitus	1.508	0.905–2.511	0.115
Chronic renal failure on hemodialysis	0.524	0.271–1.015	0.055
Chronic total occlusion	0.962	0.196–4.732	0.962
In-stent lesion	0.468	0.082–2.686	0.394
Reference diameter (every 1 mm increase)	0.377	0.222–0.638	<0.001
Lesion length (every 5 mm increase)	1.179	1.085–1.282	<0.001
Moderate to severe angulation (≥30°)	2.872	1.712–4.818	<0.001
Pre-procedural TIMI-flow grade ≤2	2.336	1.170–4.667	0.016

All variables were simultaneously adjusted in one step.

**Table 4:** Multivariate logistic regression model to find modifiable factors associated with slow flow.

Dependent variable: Slow flow (≤ TIMI-2) just after RA			
*Dependent variable*: *Slow flow*			
*Independent variables*	Odds ratio	95% confidence interval	*p* value
Primary RA strategy	0.201	0.097–0.416	<0.001
Start with RotaWire Floppy	0.609	0.321–1.156	0.129
Initial burr-to-artery ratio (every 0.1 increase)	1.570	1.311–1.879	<0.001
Mean single run time (every 1 second)	1.125	1.059–1.197	<0.001
Mean rotational speed (every 1000 rpm increase)	1.003	0.985–1.021	0.763
Systolic blood pressure just before RA (every 10 mm Hg increase)	0.864	0.780–0.958	0.006
Halfway RA	2.680	1.549–4.639	<0.001
Use of ACE inhibitors/ARBs	1.600	0.924–2.771	0.093
Use of beta blockers	1.972	1.058–3.677	0.033

All variables were simultaneously adjusted in one step.

The multivariate logistic regression analysis to investigate the association between unmodifiable/modifiable factors and slow flow is shown in Table 5. Since reference diameter was closely associated with initial burr-to-artery ratio, we made 2 models to avoid multicollinearity. In model 1, reference diameter, primary RA strategy, and systolic BP just before RA were inversely associated with slow flow. Lesion length, moderate to severe angulation (≥30°), mean single run time, halfway RA, and use of beta-blockers were significantly associated with slow flow. In model 2, initial burr-to-artery ratio was significantly associated with slow flow. Since mean single run time was significantly associated with slow flow, we replaced “mean single run time” with specific cut-off such as 10, 15, and 20 seconds in model 1. Short single session defined as ≤10 seconds (OR 0.526, 95% CI 0.230–1.206, *p* = 0.129) and defined as ≤20 seconds (OR 0.598, 95% CI 0.303–1.183, *p* = 0.140) were not significantly associated with slow flow, whereas short single session defined as ≤15 seconds (OR 0.458, 95% CI 0.271–0.776, *p* = 0.004) was inversely associated with slow flow. Since systolic BP just before RA was significantly associated with slow flow, we replaced “systolic BP just before RA” with specific cut-off such as 120, 130, 140, and 150 mmHg in model 1. Systolic BP just before RA ≥120 mmHg (OR 1.062, 95% CI 0.448–2.516, *p* = 0.892) and ≥130 mmHg (OR 0.824, 95% CI 0.442–1.536, *p* = 0.543) were not associated with slow flow, whereas systolic BP just before RA ≥140 mmHg (OR 0.501, 95% CI 0.297–0.843, *p* = 0.009) and ≥150 mmHg (OR 0.461, 95% CI 0.272–0.780, *p* = 0.004) were inversely associated with slow flow.

**Table 5:** Multivariate logistic regression model to find modifiable and unmodifiable factors associated with slow flow.

Dependent variable: Slow flow (≤ TIMI-2) just after RA			
**Model 1:** Reference diameter was included as an independent variable			
***Dependent variable*: *Slow flow***			
*Independent variables*	Odds ratio	95% confidence interval	*p* value
Unmodifiable factors			
Reference diameter (every 1 mm increase)	0.351	0.205–0.600	<0.001
Lesion length (every 5 mm increase)	1.193	1.093–1.301	<0.001
Moderate to severe angulation (≥30°)	2.054	1.171–3.601	0.012
Pre-procedural TIMI-flow grade ≤2	1.246	0.585–2.655	0.569
Modifiable factors			
Primary RA strategy	0.224	0.097–0.513	<0.001
Mean single run time (every 1 second)	1.085	1.022–1.152	0.008
Systolic blood pressure just before RA (every 10 mm Hg increase)	0.870	0.784–0.966	0.009
Halfway RA	2.027	1.130–3.635	0.018
Use of beta blockers	1.894	1.004–3.573	0.049
**Model 2:** Initial burr-to-artery ratio was included as an independent variable			
***Dependent variable*: *Slow flow***			
*Independent variables*	Odds ratio	95% confidence interval	*p* value
Unmodifiable factors			
Lesion length (every 5 mm increase)	1.198	1.099–1.305	<0.001
Moderate to severe angulation (≥30°)	1.996	1.133–3.515	0.017
Pre-procedural TIMI-flow grade ≤2	1.218	0.565–2.627	0.615
Modifiable factors			
Primary RA strategy	0.234	0.102–0.535	0.001
Initial burr-to-artery ratio (every 0.1 increase)	1.451	1.212–1.737	<0.001
Mean single run time (every 1 second)	1.117	1.051–1.187	<0.001
Systolic blood pressure just before RA (every 10 mm Hg increase)	0.875	0.788–0.972	0.013
Halfway RA	2.018	1.119–3.640	0.020
Use of beta blockers	1.923	1.017–3.635	0.044

All variables were simultaneously adjusted in one step.

## Discussion

The present study included 513 lesions treated with RA, and divided those into the slow flow (n = 97) and non-slow flow (n = 416) groups according to the slow flow just after RA. Multivariate logistic regression analysis revealed that unmodifiable factors such as lesion length and moderate to severe angulation (≥30°) were positively associated with slow flow, whereas reference diameter was inversely associated with slow flow. It would be important for accurate risk estimation to recognize these unmodifiable factors. Modifiable factors such as initial burr-to-artery ratio and use of beta blockers were positively associated with slow flow, whereas primary RA strategy, short single run (≤15 seconds), and sufficient systolic BP ≥140 mmHg just before RA were inversely associated with slow flow. It would be important for the refinement of RA procedures to understand these modifiable factors.

First, we should explain why we focused on slow flow just after RA in the present study. Slow flow/no reflow phenomenon is frequently observed during primary PCI, and typically presents as TIMI-0 or TIMI-1 flow just after stent deployment [29]. The occurrence of slow flow in primary PCI is closely associated with thrombus or lipid rich plaques [30, 31]. Unlike slow flow in primary PCI, slow flow during RA typically presents as TIMI-2 flow, and gradually worsens as TIMI-1 flow or TIMI-0 flow if RA operators do not stop RA. Thus, transient TIMI-2 flow just after RA would not necessarily results in final TIMI-2 flow or peri-procedural MI, which was confirmed by our low incidence of final TIMI-≤2 flow grade (0.8%) or peri-procedural MI with slow flow (1.9%). In other words, it would be important to manage TIMI-2 flow during RA appropriately to prevent more severe complications. In the present study, we sought to identify some characteristics to prevent slow flow and more severe later complications.

Among unmodifiable factors, reference diameter, lesion length, and moderate to severe angulation (≥30°) were significantly associated with slow flow. Reference diameter was inversely associated with slow flow, implying greater risk in the case of small vessels. Small vessel disease has been recognized as high risk for PCI, and requires RA more frequently than does non-small vessel disease [32]. Since the minimum burr size is 1.25-mm, small vessel size ≤2.0 mm would naturally have a high (≥0.6) burr-to-artery ratio, which is a known risk factor for slow flow [33]. Lesion length is also a known risk factor for slow flow during RA [13], probably because the amount of debris caused by RA would be greater in the case of diffuse long lesions than with short lesions. Moderate to severe angulation was positively associated with slow flow. In fact, angulation is closely associated with more severe complications such as perforation or burr entrapment [6, 8], partly because the shape of the burr is ellipsoid [34]. RA operators would experience difficulty in advancing the burr beyond the angle [35], which results in a long ablation time and subsequent slow flow.

Among modifiable factors, primary RA strategy, short single run (≤15 seconds), sufficient systolic BP ≥140 mmHg just before RA, initial burr-to-artery ratio, and halfway RA were significantly associated with slow flow. Primary RA strategy was inversely associated with slow flow, whereas secondary RA strategy was positively associated with it. The advantage of primary RA strategy would be the absence of coronary dissection or intimal hematoma, because balloon dilatation was not tried before RA. On the other hand, lesions with secondary RA strategy might have more complex features such as chronic total occlusion than those with primary RA strategy [36, 37]. Even after multivariate analysis, it would be difficult for our retrospective study to prove whether primary RA strategy was truly ideal for severely calcified lesions. Short single run (≤15 seconds) was inversely associated with the occurrence of slow flow. Although expert consensus documents have recommended short single run to prevent slow flow [38], no evidence was presented supporting short single run. To the best of our knowledge, our study provides the first information regarding short single run for the prevention of slow flow. Similarly, although expert consensus documents emphasize the importance of appropriate BP for the prevention of slow flow [38], there has been no evidence in support of this recommendation. Our study suggests the importance of sufficient systolic BP (≥140 mmHg) for the prevention of slow flow. Initial burr-to-artery ratio was significantly associated with slow flow, which has been described in earlier reports and expert consensus documents [33, 38, 39]. Our study confirmed the importance of conservative bur-to-artery ratio using a large database. Halfway RA was significantly associated with slow flow, probably because halfway RA was performed on more complex lesions such as diffuse long lesions or angulated lesions. Our group previously reported that the incidence of slow flow did not differ between the propensity-score matched conventional RA and the propensity-score matched halfway RA [22].

The use of beta blockers was also associated with slow flow. In 1997, Sharma et al. reported the association between use of beta blockers and slow flow among 225 PCI with RA, and speculated that vasospasm might be induced by beta blockers [13]. In 2012, our group previously examined the association between use of beta blockers and slow flow among 186 PCI with RA, and concluded that beta blockers were not associated with slow flow [19]. In the present study, we included 513 PCI with RA, checked slow flow very carefully, and run more robust multivariate analysis. However, the mechanism of slow flow in patients with beta blockers is not clear. Even in the non-slow flow group, approximately 70% of patients had beta blockers, which implies that beta blockers do not always prevent coronary flow just after RA. Since beta blockers are the cornerstone of optimal medical therapy [40], it is not realistic to stop beta blockers before PCI with RA. A prospective study is warranted to confirm whether beta blockers affect coronary flow just after RA. Although the type of RotaWire (floppy or extra-support) was significantly associated with slow flow in the univariate analysis, the association between the type of RotaWire and slow flow was not significant in the multivariate analysis. Recently, the expert consensus document on RA including guidance for selection of RotaWires was published from the Japanese association of cardiovascular intervention and therapeutics [10]. Since the ability to ablate the severely calcified plaques is greater in extra-support than in floppy [10], we might use extra-support for more complex lesions, which may explain why extra-support was associated with slow flow in the univariate analysis, but was not in the multivariate analysis.

The clinical implications of the present study should be noted. Our results have provided RA operators with high risk features, such as small diameter vessels, diffuse long lesions, and angulation. Because those factors are unmodifiable, RA operators, especially junior RA operators, need to prepare intravascular vasodilators, intravenous vasopressor to maintain BP, and rescue IABP support. Total ablation time is not a modifiable factor in RA, because diffuse long lesions would naturally require longer total ablation time. However, unlike total ablation time, single run time is a modifiable factor. RA operators can shorten single run time intentionally. Although the manufacturer recommends <30 seconds as single run time, 30 seconds would be too long for the prevention of slow flow. Because there is no drawback in short single run, we recommend that RA operators should use short single run (≤15 seconds) for the prevention of slow flow. Systolic BP was inversely associated with slow flow. Since low systolic BP might reflect poor cardiac function, it was uncertain whether systolic BP was a truly modifiable factor. However, it would be acceptable to recommend that RA operators consider using an intravenous vasopressor to maintain BP over 140 mmHg just before RA when the patient’s systolic BP is low. As initial burr-to-artery ratio was closely associated with slow flow, we should select a small burr to achieve low burr-to-artery ratio. However, since the minimum size of the burr is 1.25-mm, it may be difficult to achieve low (<0.5) burr-to-artery ratio for small diameter vessels. The importance of short single run time or sufficient systolic BP before RA would be greater for the treatment of small diameter vessels than for that of large diameter vessels.

### Study limitation

This study has the following limitations. Since our study was designed as a single-center, retrospective, observational study, there was a possibility of selection bias. Of 513 study lesions, 510 lesions (99.4%) were performed or supervised by a senior operator (K. Sakakura). Therefore, operator bias within the institution was minimal. However, the senior operator’s decision to perform RA or technique regarding RA might not be consistent during the study period over 6 years, because the senior operator himself learned and accumulated extensive experience from cases over 6 years. Slow flow might be influenced by various factors such as the settings of power injectors, the presence of side holes in guide catheters, and an unblinded evaluator (Sakakura K), which would limit the reproducibility of this study. Although we divided parameters into unmodifiable and modifiable factors in 2 multivariate logistic regression analyses, some modifiable factors such as primary RA strategy might not be modifiable, because some operators routinely adopt a strategy of primary RA. Initial burr-to-artery ratio might not be modifiable, if the reference diameter was very small. Thus, the classification of the variables into modifiable and non-modifiable might be arbitrary. The models that lumped both modifiable and non-modifiable may be more reasonable. However, since the number of slow flow was not sufficient to accommodate all variables together, we selected variables from each category to avoid overfitting of the model. Although our group reported the inability to cross the lesion with IVUS as a predictor for slow flow [41], we did not include variables from intravascular imaging devices as potential factors, because we did not try intravascular imaging before RA in not a few lesions (n = 157) in the present study. Moreover, even when we tried intravascular imaging devices, intravascular imaging devices did not cross the lesion in 164 lesions. Therefore, we focused on factors other than variables from intravascular imaging devices in the present study. The incidence of slow flow was higher in the present study than earlier studies [15, 42], partly because we captured transient mild slow flow that would not affect clinical outcomes. We did not distinguish between transient mild slow flow and severe permanent slow flow in the present study, because transient slow flow may be a warning sign for more severe complications. An expert consensus document also emphasizes the immediate management for TIMI-2 flow to prevent more severe slow flow [10].

## Conclusions

Slow flow was positively associated with several unmodifiable factors including lesion length and angulation, and inversely associated with reference diameter. In addition, slow flow was positively associated with several modifiable factors including initial burr-to-artery ratio and use of beta blockers, and inversely associated with primary RA strategy, short single run, and systolic blood pressure just before RA. Application of this information could help to improve RA procedures.

## Supporting information

S1 Dataset Dataset of all study lesions. (XLSX)
